# The association between hip fracture and hip osteoarthritis: A case-control study

**DOI:** 10.1186/1471-2474-11-274

**Published:** 2010-11-26

**Authors:** Jonas Franklin, Martin Englund, Torvaldur Ingvarsson, Stefan Lohmander

**Affiliations:** 1 University Hospital, Akureyri, Iceland; 2 Department of Orthopedics, Clinical Sciences Lund, Lund University, Sweden; 3 Clinical Epidemiology Research & Training Unit, Boston University School of Medicine, Boston, MA, USA; 4 Department of Health Sciences, University of Akureyri, Iceland and Faculty of Medicine, University of Iceland, Reykjavík, Iceland

## Abstract

**Background:**

There have been reports both supporting and refuting an inverse relationship between hip fracture and hip osteoarthritis (OA). We explore this relationship using a case-control study design.

**Methods:**

Exclusion criteria were previous hip fracture (same side or contralateral side), age younger than 60 years, foreign nationality, pathological fracture, rheumatoid arthritis and cases were radiographic examinations were not found in the archives. We studied all subjects with hip fracture that remained after the exclusion process that were treated at Akureyri University Hospital, Iceland 1990-2008, n = 562 (74% women). Hip fracture cases were compared with a cohort of subjects with colon radiographs, n = 803 (54% women) to determine expected population prevalence of hip OA. Presence of radiographic hip OA was defined as a minimum joint space of 2.5 mm or less on an anteroposterior radiograph, or Kellgren and Lawrence grade 2 or higher. Possible causes of secondary osteoporosis were identified by review of medical records.

**Results:**

The age-adjusted odds ratio (OR) for subjects with hip fracture having radiographic hip OA was 0.30 (95% confidence interval [95% CI] 0.12-0.74) for men and 0.33 (95% CI 0.19-0.58) for women, compared to controls. The probability for subjects with hip fracture and hip OA having a secondary cause of osteoporosis was three times higher than for subjects with hip fracture without hip OA.

**Conclusion:**

The results of our study support an inverse relationship between hip fractures and hip OA.

## Background

It is a common clinical observation that patients with hip fracture very rarely have hip osteoarthritis (OA)[1-3]. This has been examined in several studies, some claiming that patients with hip fracture have less hip OA than expected[4-6], others that there is no difference between hip fracture patients and the general population[7,8]. Some studies have claimed that hip OA is only protective against intracapsular fractures[9,10], while one study found that patients with hip OA have an increased risk for fracture[11]. An increased bone density in the femoral neck and a reduced density in the trochanter region in hips with OA, compared to hips without OA, was suggested to increase the risk for extracapsular fractures[12]. Low bone density increases the risk for hip fractures[13], and patients with hip OA have higher bone density in the femoral neck[14]. An inverse relationship between hip OA and hip fracture has been suggested, possibly associated with genetic variation[14,15].

The studies done so far have varied greatly in their methodology and definition of hip OA, some using self-report of hip OA[4,5,8], and others a radiographic definition[6,9-11,16]. The latter studies have in addition used different scoring systems for the definition of hip OA. Some studies have been longitudinal and others cross-sectional. The cross-sectional studies have varied in the characteristics of the control groups. All this makes interpretation of the published evidence difficult.

The purpose of the present study was to test the hypothesis that subjects with hip fracture are less likely to have radiographic hip OA than control subjects without hip fracture.

## Methods

The study was approved by the Ethics Committee of Akureyri University Hospital, where the study was based.

### Hip fracture cases

**Figure 1:**
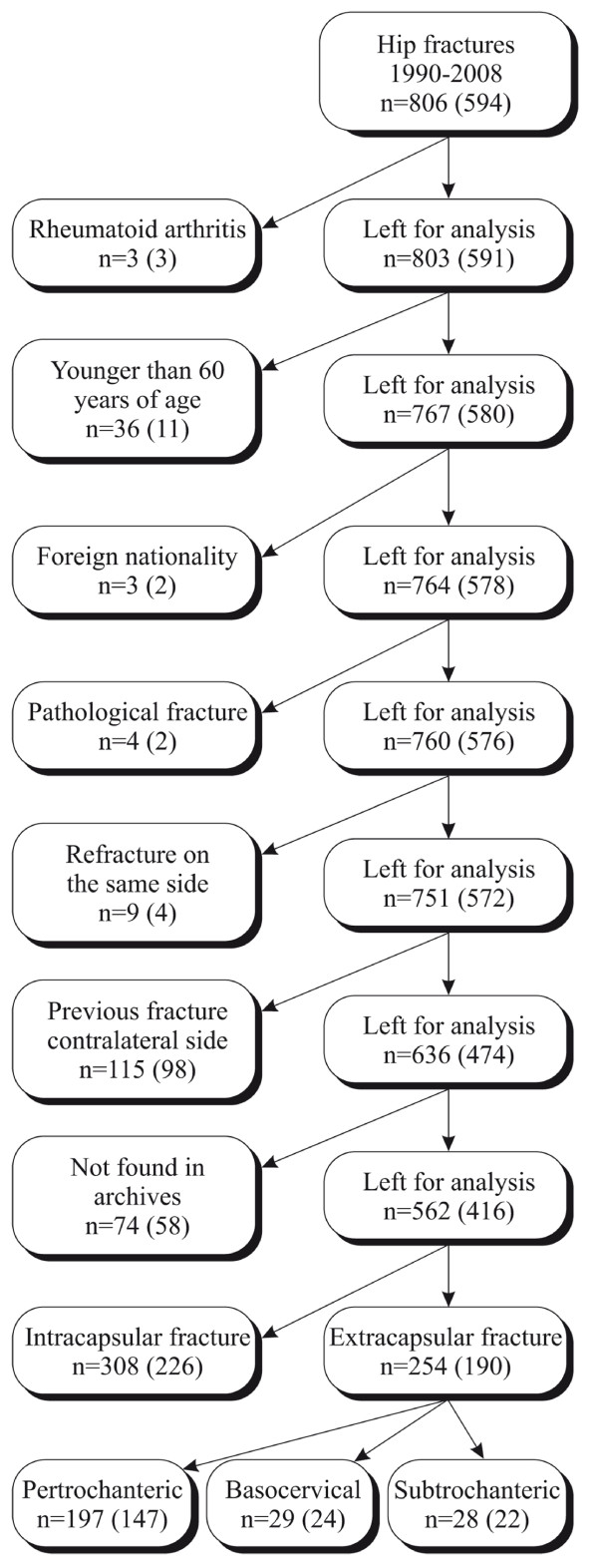
**Flow chart of recruitment of hip fracture cases**. n = the total number of subjects in each group, the number within parenthesis is the number of women in each group.

In Iceland, as in other Scandinavian countries, all persons have a unique personal identification number. This, combined with a highly computerized national health system, makes it possible to identify all patients operated on for a given diagnosis or by a certain procedure in Iceland[17]. We identified 806 consecutive cases of hip fracture that were admitted to Akureyri University Hospital during 1990-2008. This constitutes 24% of all hip fractures in Iceland during that time. Patients were from both rural and urban areas. As younger hip fracture patients are more likely to have sustained a high energy trauma and sustain fracture in the absence of osteoporosis, all patients younger than 60 years and all patients with a previous hip fracture were excluded. After exclusions (Figure 1), we had 636 eligible cases with a first time hip fracture, and 562 of these had pre-operative hip radiographs available.

### Controls with colon radiographs

Our control cohort was based on patients who had colon radiographs taken due to referral in routine health care. Colon radiographs taken at three different radiographic departments in Iceland during the years 1980-97 were examined. The patients were referred to these radiographic departments from four different hospitals (community and academic), as well as from the primary health care system. Patients were from both rural and urban areas. We excluded all subjects who were younger than 60 years of age, leaving 803 controls, of which 438 were women (54.5%). Iceland has a homogenous population of just over 300,000 inhabitants. The hip fracture patients were all treated at one hospital, but the controls came from three different hospitals. 38% of the controls were from the same hospital as the cases.

### Radiographic techniques

The double contrast (barium enema) colon radiographs included at least two anteroposterior (AP) and several oblique exposures. In this study the hip joints were assessed from a supine AP control radiograph, which was taken with the same tube to film distance of 100 cm that is used in a standard AP view of the pelvis. The x-ray beam was centered on the umbilicus. Both hips were graded and we then randomly chose the right or the left hip for our control cohort.

In our case cohort preoperative radiographs were available for 562 (88%) cases. Pelvic radiographs showing the contralateral hip were available for 457 of patients (81.3%). The hip joints were assessed from a standard supine AP pelvic radiograph, taken with a tube to film distance of 100 cm with the x-ray beam centered on the symphysis pubis. We measured hip joint space with an electronic caliper[18] and assessed osteophytes, sclerosis, cysts and any signs of secondary OA[19]. The same pre-operative radiographs were used for diagnosing the fracture and for the assessment of hip OA.

Hip fractures were classified as intra- or extracapsular[20]. We measured minimal joint space (MJS) and graded the radiographic features of OA according to Kellgren and Lawrence[19], and individual features of OA, osteophytes, sclerosis and cysts in both hips.

All radiographs of cases were examined by a single observer (JF). All controls had been previously read by a different author (TI)[21]. To calculate inter- and intraobserver reliability [22], we selected 50 radiographs of cases and 50 radiographs of controls, that included the full range of radiographic features that were read again by both these authors. We used Cohen's kappa to calculate the intra- and interobserver reliability for detecting hip OA.

For MJS both the intra- and interobserver reliability was high, but Kellgren and Lawrence grading was not as reliable (Table 1).

**Table 1:** Intra- and interobserver reliability measured by Cohen's κ

Radiographic method	Definition of disease positive	Intra-observer reliability			Inter-observer reliability		
			
		Controls	Fractured side	Contralateral side	Controls	Fractured side	Contralateral side
Minimum joint space	≤ 2.5 mm	0.94*	0.91	0.89	0.84	0.80	0.82
Kellgren and Lawrence	≥ grade 2	0.76*	0.93	0.56	0.67	0.63	0.50

* Previously published results [21]

### Validity of pre-operative radiographs

Pre-operative radiographs of cases were used since postoperative radiographs are now often taken in the operating theatre which yields radiographs that cannot be used to evaluate MJS. Presence of a hip fracture (displaced or not displaced) may affect the measurement of the joint space in the hip, even if subtle changes in position do not affect MJS in the hip[23]. To evaluate this we selected 50 patients where we had both pre- and postoperative standard pelvic radiographs, with an anatomic reduction of the fracture, and compared MJS measurement pre- and post-operatively. All the post-operative radiographs were taken within 10 days from the fracture. The 50 patients that were used for comparison of pre- and post-operative x-rays were not randomly chosen, since we were limited to patients that had a valid postoperative radiograph. We therefore tested if our sample adequately represented our fracture cases. Independent samples t-test for MJS was not significant (p = 0.96) and neither was Kolmogorov-Smirnov Z for OA/Not OA status (p = 0.36), which shows that this sample of 50 cases adequately represented our fracture cohort. There were 3 patients classified as having OA on a preoperative x-ray that were not classified as having OA on the postoperative x-ray. One patient was classified as having OA on the postoperative x-ray, that did not have OA on the preoperative x-ray. We calculated the p-value for pre- and postoperative readings of MJS with paired t-test (p = 0.18) and used the McNemars test to compare OA/Not OA status on pre- and postoperative x-rays (p = 0.63). Neither was significant, thereby validating the choice of using pre-operative x-ray examinations.

Unless otherwise stated, hip OA was defined as MJS of 2.5 mm or less[21,22]. In the hip fracture cohort there were 6 women and one man that had a total hip replacement due to OA in the contralateral hip. These were classified as having OA in that hip.

### Secondary osteoporosis

Medications or diseases that cause secondary osteoporosis and thus increase the risk for hip fracture might be more common in the case or control group. To explore the association with risk factors for secondary osteoporosis in patients with hip fracture we did a prevalence study of possible risk factors for secondary osteoporosis within the hip fracture cohort using a matched design. This method was chosen as we did not have access to data on possible causes of secondary osteoporosis for the control cohort. We defined patients with hip fracture and MJS 2.5 mm or less as hip OA cases and to each of these we assigned two reference subjects, who had hip fracture, but not having hip OA (hence both hip OA cases and reference subjects came from the hip fracture cohort). The reference subjects were matched according to gender, fracture type and age ± 1 year. If more than 2 possible reference subjects were found, we randomly chose which to use. We reviewed the medical records of hip OA cases and their matched reference subjects from the fracture cohort and registered possible causes of secondary osteoporosis that we identified [24,25].

### Statistical methods

Age differences between groups were tested with the independent samples t-test. We age standardized the observed prevalence of hip OA in both hip fracture cases and controls against the Icelandic population from the year 2000 (using 5 year age strata and direct external standardization)[26]. Intra- and interobserver reliability was calculated using Cohen's Kappa. Paired t-test and McNemar test were used to compare readings of pre- and postoperative x-rays and we used independent samples t-test and Kolmogorov-Smirnov Z to see that this not randomly selected sample had approximately the same mean MJS and distribution of values as the entire group of cases. Chi-square was used in calculations for difference in crude rate and binary logistic regression for age-adjusted calculation of odds ratios for having OA amongst cases vs. controls. We considered a p-value of less than or equal to 0.05 to be significant, and all tests were 2-tailed. Calculations were done using SPSS v. 16.0. (SPSS Incorporated 2007).

## Results

### Case and control demographics and characteristics

The male to female ratio was about 1:3 in the fracture cohort, while it was close to one in the control cohort (Table 2). The number of basocervical and subtrochanteric fractures was small, so these were grouped together with pertrochanteric fractures as extracapsular fractures (Figure 1). There was no difference in mean age between the two fracture types, neither for men (p = 0.85), nor women (p = 0.48). The mean age of controls was significantly lower than that of subjects with fracture (p < 0.0001).

**Table 2:** Demographics of cases with hip fracture and controls

	Cases (hip fractures)			Controls
		
	All	Intracapsular	Extracapsular	
Sex				
Men	146 (26.0%)	82 (26.6%)	64 (25.2%)	365 (45.5%)
Women	416 (74.0%)	226 (73.4%)	190 (74.8%)	438 (54.5%)
Age				
Men	80.3 (8.6)	80.4 (8.3)	80.1 (9.1)	70.9 (7.6)
Women	81.5 (8.1)	81.3 (8.0)	81.8 (8.2)	70.3 (7.1)

Values are number (%) or mean (SD).

### Prevalence of hip osteoarthritis

There was a significant difference in the crude prevalence of hip OA between fracture cases and controls for men (p = 0.02), but not for women (p = 0.3). Following a direct external standardization of the crude prevalence, the differences between our cases and controls increased, especially for women where the age difference between cases and controls was greater (Table 3).

**Table 3:** Prevalence of radiographic hip OA in hip fracture cases and controls

				N	Crude prevalence, % (n)		Standardized prevalence‡, %	
					
					MJS*	K & L†	MJS*	K & L†
Men								
	Cases							
		Fractured side						
			All	146	4.8% (7)	4.8% (7)	4.1%	4.1%
			Intracapsular	82	4.9% (4)	4.9% (4)	6.8%	6.8%
			Extracapsular	64	4.7% (3)	4.7% (3)	1.2%	1.2%
		Contralateral side						
			All	121	6.6% (8)	6.6% (8)	4.3%	4.3%
			Intracapsular	69	4.3% (3)	4.3% (3)	1.2%	1.2%
			Extracapsular	52	9.6% (5)	9.6% (5)	8.1%	8.1%
								
	Controls			365	11.2% (41)	11.2% (41)	11.4%	11.4%
Women								
	Cases							
		Fractured side						
			All	416	9.6% (40)	8.7% (36)	3.9%	5.5%
			Intracapsular	226	8.0% (18)	5.3% (12)	3.6%	2.7%
			Extracapsular	190	11.6% (22)	12.6% (24)	4.5%	9.4%
		Contralateral side						
			All	336	12.2% (41)	9.5% (32)	9.8%	7.8%
			Intracapsular	189	9.5% (18)	7.9% (15)	7.8%	8.0%
			Extracapsular	147	15.6% (23)	11.6% (17)	12.0%	7.8%
								
	Controls			438	11.6% (51)	10.5% (46)	13.5%	11.5%

*Osteoarthritis defined as MJS ≤ 2.5 mm. †Osteoarthritis defined as Kellgren and Lawrence grade ≥ 2. ‡ Standardized against the Icelandic population of year 2000.

### Age-adjusted association between hip fracture and hip osteoarthritis

There was no significant difference in mean age when comparing men with or without hip OA (Table 4). However, women with hip OA were significantly older than women without hip OA.

**Table 4:** Mean age according to hip OA status

	Not hip OA	Hip OA	Difference
Men			
Cases (hip fractures)	80.2 (8.6)	80.7 (9.9)	0.5 (p = 0.9)
Controls	70.7 (7.5)	72.7 (8.1)	2.0 (p = 0.1)
Difference	9.5 (p < 0.0001)	8.0 (p = 0.02)	
Women			
Cases (hip fractures)	81.1 (8.2)	86.2 (5.8)	5.1 (p < 0.001)
Controls	69.8 (6.9)	73.4 (7.4)	3.6 (p < 0.001)
Difference	11.2 (p < 0.0001)	12.7 (p < 0.0001)	

Values are mean (SD) or difference in mean age (p).

**Figure 2:**
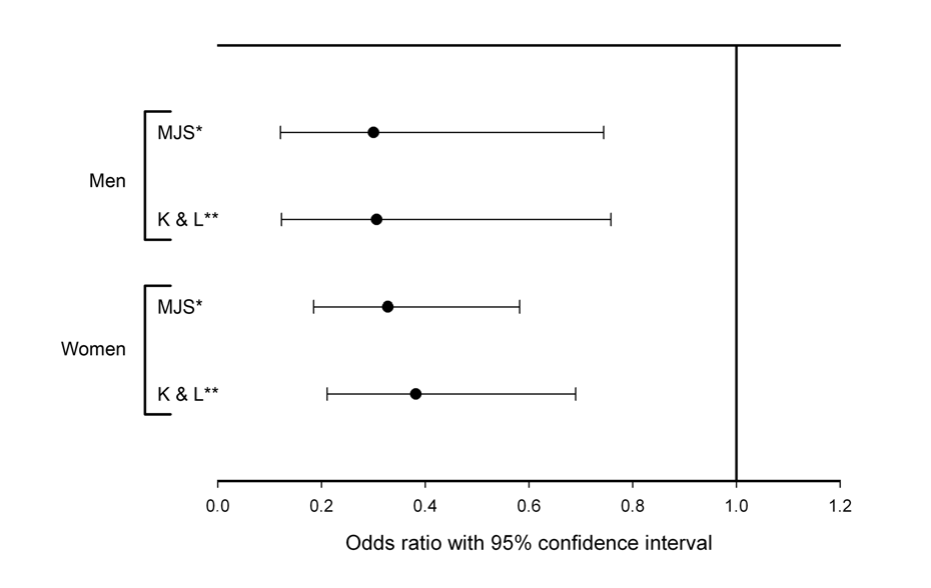
**Age adjusted odds ratio for having hip OA in the fractured hip compared to controls without hip fracture**. Error bars show 95% CI for cases compared to controls. MJS: Using hip OA defined by minimal joint space. K & L: Using hip OA defined by Kellgren and Lawrence grade.

**Figure 3:**
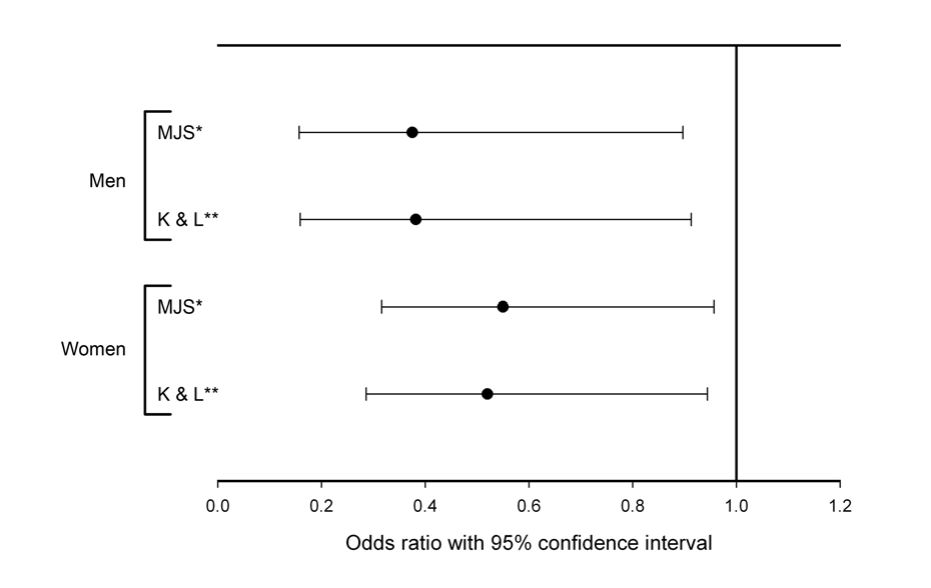
**Age adjusted odds ratio for having hip OA in the contralateral hip of hip fracture cases compared to controls without hip fracture**. Error bars show 95% CI for cases compared to controls. MJS: Using hip OA defined by minimal joint space. K & L: Using hip OA defined by Kellgren and Lawrence grade.

The age difference between cases and controls emphasized the need to take that into account when assessing the association between hip fracture and hip OA. We therefore did an age-adjusted binary logistic regression with hip OA as the dependent variable. All groups of hip fracture patients had a significantly reduced odds ratio of hip OA (comparing cases with controls) regardless of gender and classification system of hip OA. The odds ratio for hip OA was significantly reduced in both the fractured hip and the contralateral hip (Figure 2 and 3). For the fractured hip in men the OR was 0.30 (95%CI 0.12-0.74) when defining OA by MJS and 0.31 (95% CI 0.12-0.76) when defining OA by Kellgren and Lawrence. Corresponding results for the fractured hip in women were 0.33 (95%CI 0.19-0.58) and 0.38 (95% CI 0.21-0.69) when defining OA by Kellgren and Lawrence. For the contralateral hip in men the OR was 0.38 (95%CI 0.16-0.90) when defining OA by MJS and 0.38 (95% CI 0.16-0.91) when defining OA by Kellgren and Lawrence. Corresponding results for the contralateral hip in women were 0.55 (95%CI 0.32-0.96) and 0.52 (95% CI 0.29-0.94) when defining OA by Kellgren and Lawrence.

### Alternate definition of radiographic OA

In this study we have primarily used MJS 2.5 mm or less as definition of OA[21,22], but used the Kellgren and Lawrence classification as well to enable comparison to previously published studies. We also tested our data using MJS 2.0 mm or less as definition of OA. The ORs were lower (meaning a greater difference between cases and controls), although the difference from MJS 2.5 mm or less definition was not statistically significant. Using MJS 2.0 mm or less as definition the crude prevalence of OA was 9.3% for male controls, 3.1% for male cases, 8.9% for female controls and 3.7% for female cases. For hip OA in men with hip fracture, we obtained the OR 0.18 (95%CI 0.06-0.57) and in women with hip fracture the OR 0.12 (0.05-0.28).

### Intra- vs. extracapsular fractures

When defining hip OA by MJS we found no statistical difference in the age adjusted estimate of association for hip OA between the two different fracture groups, neither for men (OR = 0.96, 95% CI 0.21-4.5), or for women (OR = 1.5, 95% CI 0.75-2.8). When using Kellgren and Lawrence definition of OA, we again found no difference between the fracture types for men (OR = 0.96, 95% CI 0.21-4.5), but a significant difference for women, with extracapsular more frequently having hip OA than intracapsular fractures (OR = 2.5, 95% CI 1.2-5.2). We then repeated the calculations, using presence of OA in the contralateral hip. Again, there was no difference for men (OR = 2.3, 95% CI 0.52-10.4), regardless of classification system for OA. For women there was no difference for OA classified by MJS (OR = 1.8, 95% CI 0.92-3.5) or by Kellgren and Lawrence (OR = 1.5, 95% CI 0.73-3.2).

### Secondary osteoporosis

There were 40 women and seven men who had hip fracture and hip OA in the same fractured hip according to definition by MJS. We reviewed the medical history for all, except one woman whose patient records were not found. We thus had 46 cases with hip fracture and hip OA matched by age, gender and fracture type to 92 reference subjects with hip fracture without hip OA. Patients were defined as having possible secondary osteoporosis if they had at least one of the conditions listed in Table 5. Three of 7 men and 15 of 39 women had at least one potential cause of secondary osteoporosis (Table 5). The probability of having a possible cause of secondary osteoporosis was 3 times higher for those with hip fracture and hip OA than for those with hip fracture, but without hip OA.

**Table 5:** Number (%) of hip OA cases and reference subjects without hip OA in the hip fracture cohort with risk factors for secondary osteoporosis

	Subjects with Hip OA (hip fracture and hip OA), n = 46	Reference subjects (hip fracture, but not hip OA), n = 92
Chronic obstructive pulmonary disease	8 (17%)	2 (2%)
Polymyalgica rheumatica	3 (7%)	7 (8%)
Steroid use due to other diseases	3 (7%)	0 (0%)
Coeliac disease	0 (0%)	1 (1%)
Gastrectomy (Billroth I)	5 (11%)	3 (3%)
Alcoholism	0 (0%)	1 (1%)
Renal failure	2 (4%)	0 (0%)
Hyperparathyroidism	0 (0%)	0 (0%)
Panhypopituitarism	0 (0%)	0 (0%)
At least one of the above	18 (39%)	12 (13%)

## Discussion

The primary aim of this study was to evaluate the prevalence of hip OA in patients with hip fracture, compared to controls having had colon radiography. After adjusting for age, the odds ratio for hip OA in patients with hip fracture was found to be one-third of that in the comparison group. The difference in odds ratio between cases and controls was slightly less for the contralateral hip in women, but nevertheless significant. This suggests that the inverse relationship between hip OA and hip fracture is mainly systemic, i.e. affecting the whole patient, but contribution by a local effect in the arthritic hip cannot be excluded. The nature of the systemic effect is unknown, but a genetic factor may be involved.

For men there was no difference in age-adjusted prevalence of hip OA between those with intra- and extracapsular fractures, regardless of classification system and side. For women, there was a significantly greater prevalence of hip OA in extracapsular fractures only when defined by Kellgren and Lawrence in the fractured hip. When examining hip OA prevalence defined by MJS or when examining the contralateral hip, the difference between fracture types was not significant. We therefore conclude that there is no significant difference in hip OA prevalence when comparing intracapsular and extracapsular fractures. Our finding of a statistically significant difference for women in the fractured hip, when using Kellgren and Lawrence classification, might be a chance finding or perhaps the Kellgren and Lawrence classification system is less applicable to women, as was suggested[27].

The probability for subjects with both hip fracture and hip OA having a possible secondary cause of osteoporosis was 3 times higher than for subjects with hip fracture but without hip OA. This finding supports the possibility that many of the patients with both hip fracture and hip OA had their hip fracture due to secondary osteoporosis. This could mean that if it had been possible to adjust for risk factors for secondary osteoporosis we might have found that the inverse relationship between hip fracture and hip OA is even stronger than reported in this study. These findings need to be interpreted with some caution as no gold standard definition of secondary osteoporosis exists and we retrieved information on secondary osteoporosis noted in medical records only, as we did not have bone density measures available. Further studies are needed to explore this aspect.

Possible confounders not accounted for in the present study are body mass index (BMI) and occupation, which have been shown to be independent risk factors for hip OA[28], and use of medications, such as hormone replacement therapy and bisphosphonates, that affect osteoporosis. Another limitation is that the control group had colon radiographs but the cases pelvic radiographs. A study comparing urograms with pelvic radiographs found that joint space width was on average 10% greater on the urograms[29], while another study found no significant influence of beam angle[23]. If such an effect exists it could introduce a bias away from the null. On the other hand our results were also significant when using the Kellgren and Lawrence grading system, which is less dependent on joint space.

Patients who undergo colon radiography are not a random sample of the population. Subjects with hip OA, who are seen within health care more often, may more likely be referred to colon radiography than the background population, introducing selection bias away from the null. We do not have information on BMI. Higher BMI is linked to lower hip fracture risk[30], and higher hip OA risk[28,31]. Obesity is linked to colon cancer[32] and these subjects may more commonly undergo colon radiography, but we are not aware of any studies on the BMI of the average patient undergoing colon radiography. If our controls overall had a higher BMI than the fracture cases, then they might have had a higher risk for hip OA, compared to the background population introducing bias away from the null.

Due to the relatively long period of time that the cases and controls were sampled there could be a birth cohort effect. This might be, for example, due to the fact that BMI has increased steadily between birth cohorts. The mean birth year for our cases was 1918.5 (SD 9.7) and for our controls the mean birth year was 1922.5 (SD 7.8), so a birth cohort effect should affect our cases and controls equally.

A prerequisite for a hip fracture is a fall, which can be influenced by age, comorbidity and medications. OA increases the risk for falls[33,34], which would in contrast to the above introduce bias towards the null. The sum and direction of the aforementioned biases and any unforeseen biases is difficult to ascertain.

We used an AP pelvic radiograph of patients who were admitted to our hospital because of a hip fracture for measurement of MJS and assessment of hip OA by Kellgren and Lawrence grade. We are not aware of previous publications where MJS measurements of the fractured hip have been used. We considered the possibility that the traumatized hip would not be representative of its normal state, perhaps due to bleeding or muscle spasm. We therefore validated this method. In 46 out of 50 cases the determination of presence of OA according to MJS was the same. The fact that in 4 out of 50 cases we got a different reading is not greater than would be expected from any re-reading of a radiograph. Hip adduction-abduction has also been shown to result in a mean difference of less than 0.2 mm joint space[23], which we suggest would not significantly influence our interpretations. To evaluate the whether the relationship between hip OA and hip fracture is systemic or local to the arthritic hip we graded both the fractured hip and the contralateral hip.

The Kappa values for the fractured hip were in general acceptable and similar to previously published studies. The Kappa values for the contralateral hip were somewhat lower. The films used to evaluate reliability of the readings were chosen to include the full range of radiographic features for the fractured side, but as we used the same films for the contralateral side, the spread between different Kellgren and Lawrence grades was not even and we therefore believe that this difference may be the result of the inherent flaw of the Kappa method in such cases[35].

Definition of hip OA varies between studies. Recent studies have criticised the use of Kellgren and Lawrence classification[27], while others have shown that measurement of joint space width is reliable and reflects clinical status[36]. We used both joint space width and Kellgren and Lawrence classification of hip OA to facilitate comparison with other studies.

Data on possible secondary osteoporosis was only available for our fractured patients, ideally we would have had data on secondary osteoporosis for both cases and controls. Thus, we were not able to fully evaluate to what extent secondary osteoporosis influences our conclusions, but our results indicate that secondary osteoporosis is overrepresented amongst patients with hip fracture *and *hip OA.

Some studies suggest that an inverse relationship between OA and hip fracture exists[4-6,16,37], while and others refute it[7,8]. Most of these studies are of case-control design, while the two studies that refute this relationship are prospective cohort studies. One might therefore suggest that the evidence is stronger that there is no such relationship, even though the studies that support it are greater in number. In fact, one cannot even assume that these studies oppose one another, because of the differences in case definitions in these studies. In cohort studies the exposure is determined at the start of the study. In the case of OA, the baseline radiographic examination in a cohort study does not give an accurate estimate of the prevalence of radiographic hip OA at the time of fracture, which can be several years after the baseline examination. Reports on knee OA have also supported an inverse relationship between OA and osteoporosis[38] and a molecular basis and common pathophysiology was proposed for the inverse relationship between OA and osteoporosis[39]. A genetic component to both osteoporosis[40] and OA[41], may explain why these conditions seldom coexist.

## Conclusions

To the best of our knowledge this is the first study based on radiographically verified hip OA to quantify the risk of hip OA in patients with hip fracture, and also the first study to address the possible influence of secondary osteoporosis. We found that patients with hip fracture have one-third the risk for having hip OA in the fractured hip when compared to controls having had colon radiography. In the contralateral hip, the risk decrease was similar for men, and slightly less for women.

## Competing interests

The authors declare that they have no competing interests.

## Authors' contributions

JF, TI and SL planned the study. JF read radiographs of hip fractures, TI read radiographs of controls. JF collected data and did the statistical analysis. JF drafted the manuscript. TI, SL and ME revised the manuscript. All authors took part in analysing the findings and all authors approved the final version of the manuscript.

## Pre-publication history

The pre-publication history for this paper can be accessed here:

http://www.biomedcentral.com/1471-2474/11/274/prepub
